# Potential stressors in (prospective) physical education teachers: a comparison of different career stages

**DOI:** 10.1007/s12662-022-00804-3

**Published:** 2022-04-01

**Authors:** Fabian Pels, Ulrike Hartmann, Alina Schäfer-Pels, Birte von Haaren-Mack

**Affiliations:** 1 Department of Health and Social Psychology, Institute of Psychology, German Sport University Cologne, Am Sportpark Müngersdorf 6, 50933 Cologne, Germany; 2 Department of Child and Adolescent Psychiatry and Psychotherapy, University Hospital Ulm, Steinhövelstraße 5, 89075 Ulm, Germany

**Keywords:** School sports, Teaching, Burden, Source of stress, Demand

## Abstract

Previous studies have identified stressors in physical education (PE) teachers. However, these studies lack a comprehensive consideration of potential teaching-related stressors combined with an analysis of differences in these potential stressors between different career stages. Given that many physical education teachers suffer from stress, the purpose of the present study was to investigate potential stressors in three career stages of (prospective) physical education teachers (student teachers, pre-service teachers, teachers) in order to further develop their education in terms of stress management. The results of a survey of 723 German (prospective) physical education teachers (255 student teachers, 117 pre-service teachers, 351 teachers) showed that, overall, noise, heterogeneity of students, and inadequate curriculum were reported to be the most frequent potential stressors. When controlling for teaching hours per week, teachers, and pre-service teachers did not differ in the frequency of potential stressors. However, both teachers and pre-service teachers reported significantly less lack of facilities/equipment, pupils’ discipline problems, and lack of pupils’ motivation than student teachers, and significantly more noise than PE student teachers. Additionally, teachers reported more heterogeneity of pupils than student teachers. These findings can be explained by characteristics of the specific career stages. For practical application, it can be concluded that there is a need for coping interventions that are tailored to the stressors which are salient in a specific career phase. In future research, studies should investigate stressors in different career stages longitudinally.

## Introduction

Studies show that many physical education (PE) teachers perceive high stress (Kastrup, 2007; Kastrup, Dornseifer, & Kleindienst-Cachay, 2008; Schäfer, Pels, von Haaren-Mack, & Kleinert, 2019) and suffer from health-related consequences of stress (for an overview, see von Haaren-Mack, Schäfer, Pels, & Kleinert, 2020). In terms of mental health, for instance, PE teachers report risk of burnout (Weigelt, Lohbreier, Wunsch, Kämpfe, & Klingsiek, 2014) and burnout symptoms (e.g., emotional exhaustion; Panagopoulos, Anastasiou, & Goloni, 2014). Regarding physical health, for example, PE teachers report vocal complaints (e.g., Ubillos, Centeno, Ibanez, & Iraurgi, 2015). Given these health-related consequences of stress, it is not surprising that many PE teachers contemplate leaving their jobs as PE teachers or actually leave the PE teaching profession (Lee, 2019; Mäkelä, Hirvensalo, & Whipp, 2014; Wright & Grenier, 2019). This is a problem for the PE teachers as affected individuals but also for the school system as a whole. To address this problem, its reasons need to be understood. Therefore, the purpose of this article is to compare potential stressors in a large sample of (prospective) PE teachers in order to further develop the education of (prospective) PE teachers in terms of stress management.

### Theoretical background

According to the transactional model of stress and coping (Lazarus & Folkman, 1984, 1987), individuals are in a constant transaction between themselves and their environment. Any situation of this transaction is a potential stressor. Whether a transaction is actually stressful and, thus, produces a stressful response (e.g., negative emotion) is determined through an interaction of appraisals of the respective individual (Lazarus & Folkman, 1984, 1987). The interactions of appraisals take place within a two-fold subprocess: In the primary appraisal, the individual evaluates the relevance of a given situation for his/her well-being. The situation can be evaluated as being benign-positive, non-relevant or stressful. An appraisal is stressful when it contains thoughts of harm and loss, threat or challenge, and evokes a negative emotional response (e.g., anxiety). In the secondary appraisal, the individual evaluates his/her options (e.g., personal resources) for coping with the situation. Both appraisals interact continuously without any temporal order and determine together whether a situation is perceived as stressful. For instance, the secondary appraisal can bring the individual to the conclusion that coping is possible (e.g., the individual recognizes that social support is available), which, in turn, can change the primary appraisal (e.g., the evaluation changes from threat to challenge).

The transactional model of stress and coping (Lazarus & Folkman, 1984, 1987) was transferred and adapted to the context of teacher stress by Rudow (1994) and van Dick (1999). The final model of van Dick (1999) differs from Lazarus and Folkman (1984, 1987) in three major regards: First, the model explicitly distinguishes between potential and actual stressors in teachers. Potential stressors are observable demands of teaching in a given situation (e.g., noise, large school classes, pupils’ behavior). Whether a potential stressor becomes an actual stressor is determined through the primary appraisal of the acting individual (i.e., the teacher). An actual stressor is present when it is associated with thoughts of harm and loss, threat or challenge. Second, the model introduces general characteristics of teaching (e.g., task complexity, task transparency, task variability, responsibilities) as determinants of potential and actual stressors. Third, the model introduces personal factors of the teacher (e.g., personality, age, family situation) as determinants of primary appraisal, secondary appraisal, and coping. Both the characteristics of teaching and the personal factors of the teacher are in constant interaction. For instance, on the one hand, a specific complex situation (e.g., a conflict between two pupils) could cause insecure behavior in an inexperienced pre-service teacher and, as a result, further discipline problems for these or other pupils. An experienced teacher, on the other hand, could use specific techniques to prevent the emergence of the first conflict. Accordingly, the occurrence of potential stressors is never determined by the environment per se, but by a transaction of the environment and the individual—whether or not it is an actual stressor associated with thoughts of harm and loss, threat or challenge.

Despite its strengths, the van Dick (1999) model of teacher stress does not adequately account for the impact of a teacher’s career stage on the stress process. Although van Dick (1999) describes that teacher characteristics include the teacher’s biography and teaching experience, he does not describe concrete processes and underlying mechanisms of biography and experience in terms of stress. In contrast, the Occupational Socialization Theory (OST) in PE teachers (Lawson, 1986; Richards, Templin, & Graber, 2014; Richards, Pennington, & Sinelnikov, 2019) addresses these aspects.

According to OST, the occupational socialization of (PE) teachers consists of three phases: acculturation (during formative education before a person enters the PE profession), professional socialization (during PE university education) and organizational socialization (when entering school as a workfield) (Lawson, 1986). In the phase of professional socialization, students gain mainly knowledge and acquire skills at the university. Students in the German higher education system also complete internships in schools to gain practical insight into teaching and, depending on the degree of support they receive from the supervising teacher, to gain their own initial teaching experiences. In the federal state of North Rhine-Westphalia, for example, PE student teachers in the Bachelor’s program (BA) complete two short-term internships at school and in the Master’s program (MA) a long-term internship. When entering the phase of organizational socialization (upon completion of the MA program), PE student teachers become PE pre-service teachers for a period of 18–24 months. During this period (termed as “Vorbereitungsdienst” or formerly as “Referendariat”), the prospective teachers observe experienced teachers’ lessons, plan lessons, teach lessons, receive further education in practice-oriented seminars, and are evaluated in demonstration lessons and oral examinations. Also, they take part in conferences and have meetings with parents. Upon completion of this pre-service phase, PE teachers finally are fully skilled PE teachers. Entering the phase of organizational socialization is often perceived as a reality shock. PE pre-service teachers experience that there are differences between the content of the teacher preparation program on the one hand and the demands and challenges during teaching in school on the other hand (Richards et al., 2014, 2019). This evokes feelings of uncertainty and, accordingly, the phase of organizational socialization is said to be associated with a high perception of stress. Therefore, an investigation of stressors in PE teachers should consider different career stages.

### State of research

In a recent comprehensive systematic review of existing quantitative and qualitative studies (von Haaren-Mack et al., 2020), the most important stressors in PE teachers were examined. In this review, importance was identified by three steps of evaluating quantitative studies: (1) Assigning a rank position to each stressor in every study based on the respective mean values, (2) summarizing stressors with the same content but different terms across studies to categories of stressors, and (3) ranking the categories based on the number of studies with high rankings of the stressors within this category. As main result, the curriculum (e.g., Buttkus & Miethling, 2005; Hill & Brodin, 2004; Miethling & Sohnsmeyer, 2009), facilities/equipment (e.g., Hill & Brodin, 2004; Miethling & Brand, 2004; Sáenz-López, Almagro, & Ibánez, 2011), and pupils’ discipline (e.g., Hill & Brodin, 2004; Miethling & Sohnsmeyer, 2009; Sáenz-López et al., 2011; Stanescu, Vasiliu, & Stoicescu, 2012) were identified as the three most important stressors. Furthermore, the low status of PE (i.e., PE not being considered as important as other subjects at school) and PE teachers (i.e., lack of respect towards PE teachers) was identified as an important stressor in both the qualitative and quantitative studies of the systematic review (e.g., Blankenship & Coleman, 2009; McCaughtry, Barnard, Martin, Shen, & Kulinna, 2006; O’Sullivan, 2006; Sáenz-López et al., 2011; Washburn, Richards, & Sinelnikov, 2020). In addition, noise exposure was identified as an important potential stressor based on studies that used objective assessments (Greier, Haushofer, Pletzenauer, & Stöhr, 2009; Sá, Azevedo, Martins, & Machado, 2014).

Moreover, there is one study that examined stressors in PE pre-service teachers (Ziert, 2012). This study found that teaching as a whole is perceived as a strong stressor that seems to be more important than the parallel practice-oriented seminar (e.g., termed “Zentrum für schulpraktische Lehrerausbildung” [“Center for practical teacher training”] in North-Rhine Westphalia) the pre-service teachers have to attend. In the area of teaching, the specific stressors of curriculum (e.g., poor insight into complex curriculum), facilities/equipment (e.g., lack of equipment), physical strain (associated with fear of injury), and pupils’ discipline and motivation were identified as important. This is consistent with the findings of the systematic review on stressors in skilled PE teachers (von Haaren-Mack et al., 2020).

In summary, the existing studies show that the majority of the most important stressors among prospective PE teachers relate to the area of teaching. Furthermore, the existing studies indicate that there is a twofold research gap regarding stressors in PE teachers (von Haaren-Mack et al., 2020): First, there is a strong content-related gap, namely a lack of studies examining group differences and interpersonal differences. In more detail, previous studies mainly investigated skilled PE teachers in general or selected individual groups (e.g., investigation of first-year-teachers; Ensign, Mays Woods, & Kulinna, 2018; PE pre-service teachers; Ziert, 2012). However, according to OST (Lawson, 1986; Richards et al., 2014, 2019) in terms of stressors, it is important to consider each career stage of PE teachers on its own. In contrast, there is a lack of studies with large sample sizes that quantitatively analyze differences regarding stressors between groups with differing professional experience. In addition, previous studies lack information regarding age, gender, and teaching (e.g., amount of PE lessons per week); thus, they did not use these variables as moderators or control variables in further statistical analyses (von Haaren-Mack et al., 2020). Second, there is a methodological gap in terms of the measures used. Some measures assessed the frequency, others the perceived intensity or appraisal of stressors. Taking into account these overlapping gaps, there is a need for studies that address these limitations of previous research.

Given previous findings in terms of perceived stress (Schäfer et al., 2019) along with the assumptions of OST (Lawson, 1986; Richards et al., 2014, 2019), it appears to be particularly important to consider differences between groups of differing professional experience (i.e., different career stages) in stressors. A recent German study which is the only study that has ever soundly compared career stages of PE teacher in terms of perceived stress showed that PE pre-service teachers perceived more stress than PE student teachers (i.e., PE students who will become a teacher) and skilled PE teachers (Schäfer et al., 2019). In more detail, the study results indicated that PE pre-service teachers perceived more worries (e.g., having many worries) and tension (e.g., feeling mentally exhausted) than PE student teachers and skilled PE teachers. From a theoretical point of view, these differences were explained with the help of OST (Lawson, 1986; Richards et al., 2014, 2019). The pre-service phase can be assumed to be associated with a higher perception of stress because in this phase of organizational socialization, PE pre-service teachers have their first own teaching experiences of the daily demands of school. PE pre-service teachers perceive a reality shock as they experience that there are differences between the content of the teacher preparation program and the demands and challenges at school which are associated with uncertainty (Richards et al., 2014, 2019).

### The present study

Considering the empirical findings on stressors in skilled PE teachers (von Haaren-Mack et al, 2020) and in PE pre-service teachers (Ziert, 2012) on the one hand and given the empirical differences between career stages in perceived stress (Schäfer et al., 2019) along with the assumptions and findings of OST (Lawson, 1986; Richards et al., 2014, 2019) on the other hand, the aim of the present study was to compare a large sample of three different career stages of the German system (PE student teachers, PE pre-service teachers, skilled PE teachers) in terms of potential stressors. This comparison has a high practical relevance: First, it helps identify potential peaks in stressors across all career stages that require intervention. Second, it supports the targeted development of PE student teachers and PE pre-service teachers. For instance, surveying students can help uncover unrealistic expectations about potential stressors. If results show, for example, that PE student teachers have quite different expectations about what stressors await them, this could mean that they need to be (a) educated in advance for the actually occurring stressors, and/or that they should be educated for (b) making certain stressors occur less often than they fear (e.g., concrete preparation for scenarios [e.g., classroom management that will better prevent discipline problems from occurring]).

We hypothesize that PE pre-service teachers will report a higher frequency of stressors than either PE student teachers or PE teachers. The testing of the hypothesis will be controlled for (a) gender, (b) age, and (c) the current amount of teaching hours per week given that (a) females are likely to report more stressors than men in general (e.g., Mayor, 2015), (b) specific stressors can be assumed to be age-related (e.g., physical strain; Miethling, 2007), and (c) the current amount of teaching hours has an impact on the likelihood of the occurrence of stressors. The present study focused on eight teaching-related potential stressors, which were selected because previous studies identified these stressors as most important. Basic differences between non-teaching stressors were considered too extreme between career stages (e.g., PE pre-service teachers have to pass exams, skilled PE teachers do not) and were therefore excluded.

## Methods

### Sample

The sample consisted of 723 participants (396 males, 322 females, 5 with missing gender data) ranging from 18 to 65 years of age (mean [*M*] = 32.85, standard deviation [*SD*] = 12.43)^1^. Participants were (prospective) teachers for secondary school from three different career stages (PE student teachers, PE pre-service teachers, skilled PE teachers) from North-Rhine Westphalia, Germany. There were no specific inclusion or exclusion criteria.

The career stage group of PE student teachers consisted of 255 participants (141 males, 114 females; age: *M* = 21.65 years, *SD* = 0.50, *Min* = 18, *Max* = 32). The students were enrolled in a BA (*n* = 174) or MA (*n* = 81) program. On average, BA students were in the first or second term of their program (*M* = 1.59, *SD* = 1.27) and MA students were in the third term of their program (*M* = 3.01, *SD* = 3.27). The career stage group of PE pre-service teachers comprised 117 participants (65 males, 49 females, 3 with missing data; age: *M* = 28.53 years, *SD* = 2.78, *Min* = 25, *Max* = 43). On average, participants were in the first year of their pre-service phase (*M* = 0.98, *SD* = 0.87) and taught 6.33 PE lessons per week (*SD* = 2.02). The career stage of skilled PE teachers consisted of 351 participants (190 males, 159 females, 2 with missing data; age: *M* = 42.43 years, *SD* = 11.02, *Min* = 26, *Max* = 65). On average, they had a teaching experience of 13.06 years (*SD* = 9.65) and taught 10.06 (*SD* = 5.53) PE lessons per week.

### Measures

#### Dependent variables

Teaching-related* stressors* were assessed using the well-established German-language instrument “Arbeitsbelastungen im Sportlehrerberuf” (ABIS [“Workplace Demands in the PE Teaching Profession”]; Heim & Klimek, 1999) and two additional single items. The ABIS instrument consists of 23 items, each considering a different stressor. The items are grouped into six factors with three to five items per factor. The factors are *lack of pupil discipline* (hereafter referred to as *discipline*; four items, e.g., “Pupils disturb the PE lesson through aggressive behaviour”; α = 0.81), *inadequate facilities/equipment* (*facilities/equipment*; three items, e.g., “There is a lack of equipment for decent PE”; α = 0.82), *lack of pupil motivation* (*motivation*; five items, e.g., “Most of the pupils are absent-minded during PE”; α = 0.84), *inadequate curriculum* (*curriculum*; four items, e.g., “The content of the curriculum is overwhelming the pupils”; α = 0.73), teaching-related *problems with colleagues* (*colleagues*; four items, e.g., “Not earlier than the beginning of the lesson do I know from colleagues what teaching materials (equipment, balls, etc.) I can use.”; α = 0.71), and *physical strain* (three items, e.g., “Demonstrating exercises makes me breathe hard”; α = 0.28). Except for the factor physical strain, all factors had acceptable to good internal consistency. Despite the low internal consistency, the factor physical strain remained in the analysis because low internal consistency can be caused by the low number of items (Cortina, 1993) and because the factor has a face validity with each of the items reflecting a different aspect of physical strain. In addition to the aforementioned items and factors of the ABIS, we added two single items asking about two further stressors, namely, *pupils’ heterogeneity* (*heterogeneity*; “The heterogeneity (e.g., cultural background, physical conditions) of pupils is too high”) and *noise* (“The noise during PE is too high”), to complete the set of most important teaching-related stressors that were identified in previous literature (cf. e.g., Lautenbach, 2019; Sá et al., 2014). In sum, the final questionnaire comprised 25 items, each asking about a specific stressor. For each of the stressors, participants were asked to indicate the frequency of its actual occurrence (PE pre-service teachers and skilled PE teachers) or the anticipated frequency of its occurrence (PE student teachers) based on the existing experience and expectations, respectively, in the everyday life of a PE teacher (hereafter referred to as *frequency of occurrence* for all three career stage groups). The response options ranged from 1 (= *never*), via 2 (= *rarely*), 3 (= sometimes), 4 (= *quite often*), and 5 (= *almost always*) to 6 (= *all the time*).

#### Control variables

*Gender, age* and* teaching hours per week*—as assessed for the sociodemographic sample description—were used as control variables.

### Procedure

After gaining permission from the local ethics committee, participants were recruited. PE student teachers were contacted at university classes. They were allowed to fill in the questionnaire in class. PE pre-service teachers were contacted at seminars of institutions of teacher education or via their schools. Those who were recruited at seminars were allowed to fill in the questionnaire during seminar time; all others filled in the questionnaires at home. PE teachers were contacted via their school or during official meetings with PE teachers from other schools. Those who were recruited via their school filled in the questionnaires at home; all others answered the questionnaire during the respective meeting. It took approximately 30 min to answer the questionnaire. Participants did not receive any benefit from participation. The whole project was started and completed from autumn 2016 to summer 2017, i.e., prior to the coronavirus pandemic.

### Data analysis

Data were analyzed using SPSS Statistics 27 software (IBM, Armonk, NY, USA). In a preliminary step, the dataset was prepared for the main analysis by checking data plausibility, missing data, normality distributions, and multivariate outliers. All items showed less than 5% of missing data; therefore, missing data were not replaced and it can be assumed that any procedure would lead to the same results (Tabachnick & Fidell, 2014). Visual inspection of histograms revealed an approximation to normality for all dependent variables, but significant Kolmogorov–Smirnov tests (K‑S tests) did not. Given the large sample size of the present study, normality could be assumed despite the significant K‑S test: first, because K‑S tests are highly affected by the sample size with large samples facilitating undesired significant findings; second, because of the Central Limit Theorem (Tabachnick & Fidell, 2014). Multivariate outlier analyses identified six outliers. When excluding these outliers from the dataset for the subsequent main analyses, this did not result in any serious changes of the results. The six outliers were therefore left in the dataset for the main analyses, also to account for the fact that we ran epidemiologic analyses that should include the full range of individuals assessed, if possible.

The subsequent main analyses were twofold: First, descriptive statistics were run to identify how often the stressors occurred. Second, inferential statistics were run to identify differences between career stages in the (anticipated) occurrence of stressors. These differences were analyzed by an overall Multivariate Analysis of Variances (MANOVA) with eight dependent variables (DV; discipline, facilities/equipment, motivation, curriculum, colleagues, physical load, heterogeneity, noise) and one three-level independent variable (IV; career stage: PE student teachers^2^, PE pre-service teachers, skilled PE teachers). In order to further investigate each DV separately, Roy-Bargmann Stepdown Analysis were conducted because pooled within-group correlations were different from zero (Tabachnick & Fidell, 2014). Roy-Bargmann Stepdown Analysis involved three steps (Finch, 2007): First, the DVs were a‑priori placed in a descending order of theoretical and practical importance. In our analysis, the order (1. curriculum, 2. facilities/equipment, 3. discipline, 4. colleagues, 5. physical strain, 6. motivation, 7. heterogeneity, 8. noise) was defined based on the findings of the systematic review by von Haaren-Mack et al. (2020) which showed the importance of different stressors. Second, an ANOVA was run involving the DV that was ranked highest (curriculum) and the IV (career stage). Third, this DV served as a covariate in a subsequent ANCOVA which aimed at investigating the DV that was ranked second highest (facilities/equipment). The third step was continued for each DV in the defined order, with DVs at a higher rank serving as covariates for the analysis of lower ranked DVs. For each of these eight AN(C)OVAs, the significance level had to be manually Bonferroni-adjusted to α = 0.05/8 = 0.006. Pairwise post hoc comparisons between career stages within these AN(C)OVAs were automatically Bonferroni-adjusted using the respective SPSS function.

Subsequently, the MANOVA results were controlled for potentially confounding variables. Therefore, the aforementioned steps were repeated three times by additionally separately including gender (as a second IV; i.e., 3 × 2 MANOVA), age (as a covariate; i.e., MANCOVA) and number of PE teaching hours per week (as a covariate; i.e., MANCOVA), each as a control variable. Running these additional MAN(C)OVAs is in line with the recommendations by Tabachnick and Fidell (2014), saying that covariates hold the impact of potentially confounding variables constant. When analyzing the MAN(C)OVAs for potentially confounding variables, potential interaction effects between career stages and covariates were not taken into account to avoid an interpretation of statistical random findings. DVs were again further examined with the Roy-Bargmann Stepdown Analysis. In order to further analyze the significant effects of covariates in any of the aforementioned (M)ANCOVAs, bivariate correlations between stressors, age, and number of PE teaching hours per week were calculated. When including the covariate number of PE teaching hours per week, only two groups (PE pre-service teachers and PE teachers) were analyzed because students because students do not teach on a daily basis (except for some parts of internships).

## Results

### Overall prevalence of stressors

PE teachers of different career stages reported all stressors with a moderate frequency on average (Table 1). Noise was the stressor that occurred most frequently, followed by heterogeneity of pupils and curriculum. The occurrence of most of the stressors was reported “sometimes” or less often (i.e., value 3 or lower; curriculum 58.9%, facilities/equipment 58.9%, discipline 66.7%, colleagues 65.7%, physical strain 86.6%, motivation 83.4%, heterogeneity 64.7%, noise 66.5%). Only a small part of the whole sample reported the occurrence of the stressors “quite often” or more often (i.e., values higher than 4; curriculum 0.7%, facilities/equipment 2.8%, discipline 0.4%, colleagues 0.7%, physical strain < 0.1%, motivation 0.3%, heterogeneity 3.2%, noise 3.6%).

**Table 1:** Descriptive statistics of stressors

	Curriculum				Facilities/ equipment				Discipline				Colleagues				Physical strain				Motivation				Heterogeneity				Noise			
	*M*	*SD*	*Min*	*Max*	*M*	*SD*	*Min*	*Max*	*M*	*SD*	*Min*	*Max*	*M*	*SD*	*Min*	*Max*	*M*	*SD*	*Min*	*Max*	*M*	*SD*	*Min*	*Max*	*M*	*SD*	*Min*	*Max*	*M*	*SD*	*Min*	*Max*
PE student teachers	3.04	0.63	1.75	5.00	3.30	0.94	1.00	6.00	3.27	0.65	1.25	5.50	3.11	0.74	1.25	5.50	2.40	0.68	1.00	5.33	2.96	0.79	1.40	5.20	2.99	1.26	1.00	6.00	3.06	1.06	1.00	6.00
PE pre-service teachers	2.88	0.82	1.00	5.25	2.45	1.15	1.00	6.00	2.30	0.85	1.00	5.50	2.66	0.83	1.00	5.25	2.36	0.61	1.00	4.50	1.94	0.61	1.00	4.20	2.75	1.22	1.00	6.00	2.79	1.03	1.00	6.00
PE teachers	3.02	0.78	1.00	5.75	2.82	1.14	1.00	6.00	2.56	0.81	1.00	5.75	2.74	0.78	1.00	5.25	2.54	0.64	1.00	5.33	1.96	0.39	1.00	3.40	3.25	1.25	1.00	6.00	3.41	1.21	1.00	6.00
Total	3.01	0.74	1.00	5.75	2.93	1.12	1.00	6.00	2.77	0.85	1.00	5.75	2.86	0.79	1.00	5.50	2.46	0.65	1.00	5.33	2.31	0.77	1.00	5.20	3.08	1.26	1.00	6.00	3.19	1.16	1.00	6.00

### Differences in stressors between career stages

In order to identify differences in the occurrence of stressors between PE teachers of different career stages, a MANOVA was conducted. Results show that, in general, the career stages differed across all stressors (*F*(16, 1428) = 34.70, *p* < 0.001, η^2^ = 0.28). In order to control for potential biases due to *gender, age*, and *teaching hours*, the analysis was counterchecked with a separate MANOVA including *gender* and two separate MANCOVAs including *age* and *teaching hours* per week as covariates. When including *gender*, the main effect of career stages remained (*F*(16, 1412) = 34.01, *p* < 0.001, η^2^ = 0.28) and there was a main effect of *gender* (*F*(8, 705) = 3.15, *p* = 0.002, η^2^ = 0.04). In the MANCOVA including *age*, the main effect of career stages remained, too (*F*(16, 1426) = 21.60, *p* < 0.001, η^2^ = 0.20), and there was a main effect of *age* as well (*F*(8, 712) = 5.74, *p* < 0.001, η^2^ = 0.06). In the MANCOVA including *teaching hours*, again, the main effect of career stages (i.e., PE teachers and PE pre-service teachers) remained (*F*(1, 456) = 3.27, *p* = 0.001, η^2^ = 0.06), but there was no effect of *teaching hours* (*F*(8, 449) = 1.76, *p* = 0.084, η^2^ = 0.03).

In order to further analyze the global effects of the MAN(C)OVAs, AN(C)OVAs were conducted, each analyzing one specific stressor (Table 2).

**Table 2:** Summary of AN(C)OVA and post hoc test results for hypothesis testing (differences between career stages in stressors)

		AN(C)OVA					AN(C)OVA (incl. gender)					ANCOVA (incl. age)					ANCOVA (incl. Teaching hours)				
DV	Effect	*df1*	*df2*	*F*	*p*	η^2^*/d*	*df1*	*df2*	*F*	*p*	η^2^*/d*	*df1*	*df2*	*F*	*p*	η^2^*/d*	*df1*	*df2*	*F*	*p*	η^2^*/d*
Curriculum	Group	2	720	2.08	0.125	0.01	2	712	2.77	0.063	0.01	2	719	3.62	0.027	0.01	1	456	0.92	0.337	< 0.01
	Post hoc	Teachers > pre-service teachers				0.18	Teachers > pre-service teachers				0.22	Teachers > pre-service teachers				0.03	Teachers > pre-service teachers				0.11
	Post hoc	Teachers < student teachers				0.03	Teachers < student teachers				0.05	Teachers < student teachers				0.20	–				
	Post hoc	Pre-service teachers < student teachers				0.24	Pre-service teachers < student teachers				0.27	Pre-service teachers < student teachers *				0.24	–				
	Gender	–	–	–	–	–	1	712	4.66	0.031	0.01	–	–	–	–	–	–	–	–	–	–
	Age	–	–	–	–	–	–	–	–	–	–	1	719	5.16	0.023	0.01	–	–	–	–	–
	Teaching hours	–	–	–	–	–	–	–	–	–	–	–	–	–	–	–	1	456	2.99	0.084	0.01
Facilities/ equipment	Group	2	719	27.74	< 0.001	0.07	2	711	26.16	< 0.001	0.07	2	718	17.45	< 0.001	0.05	1	455	6.20	0.013 ^a^	0.01
	Post hoc	Teachers > pre-service teachers *				0.30	Teachers > pre-service teachers *				0.28	Teachers > pre-service teachers ***				0.42	Teachers > pre-service teachers				0.28
	Post hoc	Teachers < student teachers ***				0.47	Teachers < student teachers ***				0.45	Teachers < student teachers				0.11	–				
	Post hoc	Pre-service teachers < student teachers ***				0.76	Pre-service teachers < student teachers ***				0.70	Pre-service teachers < student teachers ***				0.53	–				
	Gender	–	–	–	–	–	1	711	4.86	0.028	0.01	–	–	–	–	–	–	–	–	–	–
	Age	–	–	–	–	–	–	–	–	–	–	1	718	10.96	< 0.001	0.02	–	–	–	–	–
	Teaching hours	–	–	–	–	–	–	–	–	–	–	–	–	–	–	–	1	455	0.02	0.886	< 0.01
Discipline	Group	2	718	75.81	< 0.001	0.17	2	710	75.27	< 0.001	0.18	2	717	49.35	< 0.001	0.12	1	454	3.39	0.066	0.01
	Post hoc	Teachers > pre-service teachers				0.25	Teachers > pre-service teachers				0.21	Teachers > pre-service teachers				0.23	Teachers > pre-service teachers				0.21
	Post hoc	Teachers < student teachers ***				0.90	Teachers < student teachers ***				0.92	Teachers < student teachers ***				0.66	–	–	–	–	–
	Post hoc	Pre-service teachers < student teachers ***				1.14	Pre-service teachers < student teachers ***				1.12	Pre-service teachers < student teachers ***				0.92	–	–	–	–	–
	Gender	–	–	–	–	–	1	710	1.34	0.248	< 0.01	–	–	–	–	–	–	–	–	–	–
	Age	–	–	–	–	–	–	–	–	–	–	1	717	0.258	0.612	< 0.01	–	–	–	–	–
	Teaching hours	–	–	–	–	–	–	–	–	–	–	–	–	–	–	–	1	454	1.41	0.236	< 0.01
Colleagues	Group	2	717	5.42	0.005	0.02	2	709	5.29	0.005	0.02	2	716	1.37	0.256	< 0.01	1	453	0.30	0.584	< 0.01
	Post hoc	Teachers < pre-service teachers				0.02	Teachers < pre-service teachers				0.03	Teachers > pre-service teachers				0.10	Teachers < pre-service teachers				0.06
	Post hoc	Teachers < student teachers **				0.28	Teachers < student teachers **				0.28	Teachers < student teachers				0.06	–	–	–	–	–
	Post hoc	Pre-service teachers < student teachers				0.26	Pre-service teachers < student teachers				0.24	Pre-service teachers < student teachers				0.16	–	–	–	–	–
	Gender	–	–	–	–	–	1	709	1.33	0.249	< 0.01	–	–	–	–	–	–	–	–	–	–
	Age	–	–	–	–	–	–	–	–	–	–	1	716	5.22	0.023	0.01	–	–	–	–	–
	Teaching hours	–	–	–	–	–	–	–	–	–	–	–	–	–	–	–	1	453	1.34	0.248	< 0.01
Physical strain	Group	2	716	9.40	< 0.001	0.03	2	708	9.28	<0.001	0.03	2	715	0.20	0.818	< 0.01	1	452	6.14	0.014	0.01
	Post hoc	Teachers > pre-service teachers				0.23	Teachers > pre-service teachers				0.21	Teachers < pre-service teachers				0.06	Teachers > pre-service teachers				0.28
	Post hoc	Teachers > student teachers ***				0.36	Teachers > student teachers ***				0.36	Teachers < student teachers				0.04	–	–	–	–	–
	Post hoc	Pre-service teachers > student teachers				0.14	Pre-service teachers > student teachers				0.16	Pre-service teachers > student teachers				0.02	–	–	–	–	–
	Gender	–	–	–	–	–	1	708	12.45	<0.001	0.02	–	–	–	–	–	–	–	–	–	–
	Age	–	–	–	–	–	–	–	–	–	–	1	715	21.53	< 0.001	0.03	–	–	–	–	–
	Teaching hours	–	–	–	–	–	–	–	–	–	–	–	–	–	–	–	1	452	2.26	0.134	< 0.01
Motivation	Group	2	715	151.32	< 0.001	0.30	2	707	149.91	< 0.001	0.30	2	714	84.35	< 0.001	0.19	1	451	1.79	0.182	< 0.01
	Post hoc	Teachers < pre-service teachers				0.12	Teachers < pre-service teachers				0.12	Teachers < pre-service teachers				0.06	Teachers < pre-service teachers				0.15
	Post hoc	Teachers < student teachers ***				1.50	Teachers < student teachers ***				1.51	Teachers < student teachers ***				1.12	–	–	–	–	–
	Post hoc	Pre-service teachers < student teachers ***				1.36	Pre-service teachers < student teachers ***				1.37	Pre-service teachers < student teachers ***				1.11	–	–	–	–	–
	Gender	–	–	–	–	–	1	707	0.17	0.684	< 0.01	–	–	–	–	–	–	–	–	–	–
	Age	–	–	–	–	–	–	–	–	–	–	1	714	0.72	0.398	< 0.01	–	–	–	–	–
	Teaching hours	–	–	–	–	–	–	–	–	–	–	–	–	–	–	–	1	451	0.41	0.522	< 0.01
Heterogeneity	Group	2	714	12.94	< 0.001	0.04	2	706	12.22	< 0.001	0.03	2	713	6.35	0.002	0.02	1	450	4.03	0.045	< 0.01
	Post hoc	Teachers > pre-service teachers *				0.24	Teachers > pre-service teachers				0.22	Teachers > pre-service teachers				0.19	Teachers > pre-service teachers				0.23
	Post hoc	Teachers > student teachers ***				0.46	Teachers > student teachers ***				0.46	Teachers > student teachers **				0.36	–	–	–	–	–
	Post hoc	Pre-service teachers < student teachers				0.23	Pre-service teachers > student teachers				0.25	Pre-service teachers > student teachers				0.20	–	–	–	–	–
	Gender	–	–	–	–	–	1	706	< 0.01	0.948	< 0.01	–	–	–	–	–	–	–	–	–	–
	Age	–	–	–	–	–	–	–	–	–	–	1	713	0.09	0.765	< 0.01	–	–	–	–	–
	Teaching hours	–	–	–	–	–	–	–	–	–	–	–	–	–	–	–	–	450	1.17	0.279	< 0.01
Noise	Group	2	713	22.37	< 0.001	0.06	2	705	22.25	< 0.001	0.06	2	712	16.39	< 0.001	0.04	1	449	2.95	0.087	< 0.01
	Post hoc	Teachers > pre-service teachers **				0.34	Teachers > pre-service teachers **				0.33	Teachers > pre-service teachers **				0.34	Teachers > pre-service teachers				0.20
	Post hoc	Teachers > student teachers ***				0.61	Teachers > student teachers ***				0.61	Teachers > student teachers ***				0.58	–	–	–	–	–
	Post hoc	Pre-service teachers > student teachers*				0.29	Pre-service teachers > student teachers*				0.29	Pre-service teachers > student teachers*				0.27	–	–	–	–	–
	Gender	–	–	–	–	–	1	705	0.28	0.598	< 0.01	–	–	–	–	–	–	–	–	–	–
	Age	–	–	–	–	–	–	–	–	–	–	1	712	1.31	0.253	< 0.01	–	–	–	–	–
	Teaching hours	–	–	–	–	–	–	–	–	–	–	–	–	–	–	–	1	449	4.36	0.037	0.01

Significance level of each AN(C)OVA was manually Bonferroni-adjusted to α = 0.05/8 = 0.006 because these were follow-up analyses to the MAN(C)OVAs. Thus, only *p*-values < 0.006 were considered significant. Post hoc comparisons were automatically Bonferroni-adjusted, with *p*-values < 0.05 indicating significant differences. Effect size for AN(C)OVAs is partial η^2^ and effect size for *t*-test based post hoc tests is Cohen’s *d *(incl. consideration of effects of covariates, if applicable)

*p < 0.05, **p < 0.01, ***p < 0.001

^a^Nonsignificant result due to manual Bonferroni adjustment

#### Curriculum.

The occurrence of an inadequate curriculum did not differ between PE teachers of different career stages (*F*(2, 720) = 2.08, *p* = 0.125, η^2^ = 0.01). When including *gender* and *teaching hours* per week (i.e., teaching hours of PE teachers and PE pre-service teachers; exclusion of PE students), there were still no effects (gender: *F*(2, 712) = 2.77, *p* = 0.063, η^2^ = 0.01; teaching hours: *F*(1, 456) = 0.92, *p* = 0.337, η^2^ < 0.01). When including *age*, there was also no effect of career stages (*F*(2, 719) = 3.62, *p* = 0.027, η^2^ = 0.01).

#### Facilities/equipment.

The occurrence of inadequate facilities and equipment significantly differed between PE teachers of different career stages (*F*(2, 719) = 27.74, *p* < 0.001, η^2^ = 0.07). Post hoc tests revealed that PE student teachers reported the occurrence of the stressor more frequently than PE teachers (*p* < 0.001, *d* = 0.47) and PE pre-service teachers (*p* < 0.001, *d* = 0.76). PE teachers also reported the occurrence of the stressor more frequently than PE pre-service teachers (*p* = 0.017, *d* = 0.30).

However, when including *age*, an effect of age appeared (*F*(1, 718) = 10.96, *p* < 0.001, η^2^ = 0.02). *Age* was negatively associated with the occurrence of facilities and equipment (Table 3). Additionally, post hoc tests revealed that PE student teachers no longer differed from PE teachers (*p* = 0.791, *d* = 0.11). When including *teaching hours,* the difference between PE student teachers and PE teachers disappeared, too (*F*(1, 455) = 6.20, *p* = 0.013, η^2^ = 0.01).

**Table 3:** Correlations between study variables

	(1)	(2)	(3)	(4)	(5)	(6)	(7)	(8)	(9)
(1) Age	–	–	–	–	–	–	–	–	–
(2) Teaching hours	0.12*	–	–	–	–	–	–	–	–
(3) Curriculum	0.05	0.10*	–	–	–	–	–	–	–
(4) Facilities/equipment	−0.17***	0.07	0.33***	–	–	–	–	–	–
(5) Discipline	−0.25***	0.12**	0.35***	0.34***	–	–	–	–	–
(6) Colleagues	−0.19***	0.09	0.31***	0.26***	0.34***	–	–	–	–
(7) Physical strain	0.19***	−0.01	0.17***	0.05	0.12**	0.19***	–	–	–
(8) Motivation	−0.42***	0.08	0.32***	0.27***	0.54***	0.37***	0.08*	–	–
(9) Heterogeneity	0.11**	0.14**	0.37***	0.23***	0.30***	0.19***	0.17***	0.14***	–
(10) Noise	0.14***	0.18***	0.34***	0.15***	0.31***	0.26***	0.39***	0.21***	0.42***

*p < 0.05, **p < 0.01, ***p < 0.001

#### Discipline.

The occurrence of discipline problems significantly differed between PE teachers of different career stages (*F*(2, 718) = 75.81, *p* < 0.001, η^2^ = 0.17). In detail, Bonferroni-adjusted post hoc tests revealed that PE student teachers reported the occurrence of the stressor more frequently than PE teachers (*p* < 0.001, *d* = 0.90) and PE pre-service teachers (*p* < 0.001, *d* = 1.14). There was no difference between PE teachers and PE pre-service teachers (*p* = 0.061, *d* = 0.25).

#### Colleagues.

The occurrence of problems with colleagues significantly differed between PE teachers of different career stages (*F*(2, 717) = 5.42, *p* = 0.005, η^2^ = 0.02). As indicated by post hoc tests, PE student teachers reported the occurrence of the stressor more frequently than PE teachers (*p* = 0.004, *d* = 0.28). PE pre-service teachers did not differ in the occurrence of the stressor from PE teachers (*p* > 0.999, *d* = 0.02) or from PE student teachers (*p* = 0.085, *d* = 0.26).

However, when including *age*, the effect of career stages disappeared (*F*(2, 716) = 1.37, *p* = 0.256, η^2^ < 0.01).

#### Physical strain.

The occurrence of physical strain significantly differed between PE teachers of different career stages (*F*(2, 716) = 9.40, *p* < 0.001, η^2^ = 0.03). Post hoc tests showed that PE teachers experienced the occurrence of physical strain significantly more frequently than PE student teachers (*p* < 0.001, *d* = 0.36). PE pre-service teachers did not differ in the occurrence of the stressor from PE student teachers (*p* = 0.659, *d* = 0.14) or from PE teachers (*p* = 0.101, *d* = 0.23).

When including *gender*, there was a significant effect of gender (*F*(1, 708) = 12.45, *p* < 0.001, η^2^ = 0.02). Women reported the occurrence of physical strain more frequently than men. When including *age*, the effect of career stages disappeared (*F*(2, 715) = 0.20, *p* = 0.818, η^2^ < 0.01), indicating that the effect had occurred in the MANOVA due to age differences. A significant effect of age appeared as well (*F*(1, 715) = 21.53, *p* < 0.001, η^2^ = 0.03). Age was positively associated with the occurrence of physical strain (Table 3).

#### Motivation.

The occurrence of the lack of pupil motivation significantly differed between PE teachers of different career stages (*F*(2, 715) = 151.32, *p* < 0.001, η^2^ = 0.30). Post hoc tests showed that PE student teachers experienced the occurrence of the stressor more frequently than PE teachers (*p* < 0.001, *d* = 1.50) and PE pre-service teachers (*p* < 0.001, *d* = 1.36). However, PE teachers and PE pre-service teachers did not differ in the perception of motivation (*p* = 0.754, *d* = 0.12).

#### Heterogeneity.

The occurrence of heterogeneity significantly differed between PE teachers of different career stages (*F*(2, 714) = 12.94, *p* < 0.001, η^2^ = 0.04). PE teachers experienced heterogeneity more frequently than PE student teachers (*p* < 0.001, *d* = 0.46) and PE pre-service teachers (*p* = 0.047, *d* = 0.24). There was no difference between PE student teachers and PE pre-service teachers (*p* = 0.124, *d* = 0.23).

When including *gender,* the difference between PE teachers and PE pre-service teachers disappeared (*p* = 0.091, *d* = 0.22). PE teachers still differed from PE student teachers (*p* < 0.001, *d* = 0.46). When including age, the difference between PE teachers and PE pre-service teachers disappeared as well (*p* = 0.171, *d* = 0.19). When including *teaching hours,* the difference between PE teachers and PE pre-service teachers disappeared, too (*F*(1, 450) = 4.03, *p* = 0.045, η^2^ < 0.01).

#### Noise.

The occurrence of noise significantly differed between PE teachers of different career stages (*F*(2, 713) = 22.37, *p* < 0.001, η^2^ = 0.06). Post hoc tests revealed that PE teachers reported the occurrence of noise more frequently than PE student teachers (*p* < 0.001, *d* = 0.61) and PE pre-service teachers (*p* = 0.002, *d* = 0.34). Additionally, PE pre-service teachers reported noise more frequently than PE student teachers (*p* = 0.036, *d* = 0.29).

However, when including *teaching hours*, the difference between PE teachers and PE pre-service teachers disappeared (*F*(1, 449) = 2.95, *p* = 0.087, η^2^ < 0.01).

## Discussion

The purpose of the present study was to examine the frequency of potential stressors in a large sample of (prospective) PE teachers of different career stages. In terms of overall findings, noise, heterogeneity of pupils, and inadequate curriculum were reported to be the most frequent potential stressors. With regard to a comparison of career stages, the results indicated that there were differences among the subgroups for all stressors, except for inadequate curriculum. In detail, at first sight, PE teachers reported a higher frequency of potential stressors than PE pre-service teachers. However, all significant differences between these two groups disappeared when controlling for teaching hours per week. Thus, the more PE lessons the PE (pre-service) teachers taught, the higher was the reported frequency of potential stressors—irrespective of the career stage or the specific stressor. The comparisons between PE teachers and PE pre-service teachers on the one hand and PE student teachers on the other hand each revealed similar patterns: Both PE teachers and PE pre-service teachers reported a significantly lower frequency of lack of facilities/equipment, pupil discipline problems, and lack of pupil motivation than PE student teachers, and a significantly higher frequency of noise than PE student teachers. Additionally, only PE teachers reported a significantly lower frequency of problems with colleagues and a significantly higher frequency of physical strain and heterogeneity of pupils than PE student teachers. However, the differences in terms of problems with colleagues and physical strain disappeared when controlling for age. With increasing age, fewer problems with colleagues and more physical strain were reported. Gender did not affect any of the differences. Within the group of PE student teachers, there were no differences between BA and MA students except for motivation (with BA students reporting higher frequency of lack of pupil motivation than MA students), as identified by additional control analyses.

In terms of the most frequent potential stressors across all career stages, our results correspond with existing findings and amend these. In a previous review (von Haaren-Mack et al., 2020), inadequate curriculum (as indicated by a number of quantitative self-report studies) and noise (as indicated by studies objectively assessing noise exposure) were also found to be highly important stressors. This might be due to the fact that both inadequate curriculum and noise are only partly controllable by PE teachers (cf. goodness-of-fit-hypothesis; Forsythe & Compas, 1987). Therefore, (prospective) PE teachers should learn acceptance-based coping strategies to manage these stressors. Additionally, we found that heterogeneity of pupils was rated as a frequent stressor. It can be concluded that heterogeneity is still an issue that is reported as potentially stressful which was already found by Lautenbach (2019). On the one hand, it is nevertheless very important to highlight the societal value of heterogeneity in the academic education of (prospective) PE teachers (Hutzler, Meier, Reuker, & Zitomer, 2019). On the other hand, given that PE teachers reported dealing with heterogeneity (of pupils) to be stressful in previous studies (Lautenbach, 2019), perceived barriers to heterogeneity have to be taken seriously. To reduce these barriers, it is necessary to enhance PE teachers’ attitudes and resources (e.g., self-efficacy) towards heterogeneity (Patey, Jin, Ahn, Lee, & Yi, 2019) and to consider heterogeneity in didactic methods of PE teaching.

The frequently reported potential stressors (in particular, inadequate curriculum, noise, heterogeneity) across all career stages need to be taken seriously because they may be reasons for the development of severe health consequences. Frequent confrontation with these potential stressors can lead to an impairment of physical and mental well-being (Heim & Klimek, 1999; Miethling & Brand, 2004), especially when they become actual psychological stressors due to negative appraisal. Accordingly, a recent meta-analysis shows that PE teachers partly report very high burnout levels (Alsalhe, Chalghaf, Guelmami, Azaiez, & Bragazzi, 2021).

Our findings regarding the differences between the career stages in perceived frequency of stressors are in contrast with our hypothesis. Based on previous results in terms of perceived stress (Schäfer et al., 2019) and based on the OST in PE teachers (Lawson, 1986; Richards et al., 2014, 2019), we had hypothesized that PE pre-service teachers will report a higher frequency of stressors than PE student teachers and PE teachers. However, PE pre-service teachers did not report any potential stressor more often than PE teachers, and PE pre-service teachers only reported noise more often than PE student teachers. Where further significant differences existed between PE pre-service teachers on the one hand and PE teachers and PE student teachers on the other, the latter two each reported higher scores. But when controlling for teaching hours per week, PE teachers and PE pre-service teachers did not differ in the frequency of potential stressors any more. These findings can be explained by characteristics of the specific career stages but also by methodological aspects of our study.

First and foremost, the partly significantly higher frequency of potential stressors in PE teachers (compared to PE pre-service teachers) can be explained by the teachers’ higher number of PE teaching per week. The corresponding effect of the respective covariate was statistically significant. Because (PE) pre-service teachers generally have fewer hours of teaching (e.g., because they also have to visit the seminar), they are less exposed to the potential stressors. Accordingly, there is a lower likelihood that potential stressors occur.

However, there are two additional reasons by which the low frequency of potential stressors among PE pre-service teachers can be explained. On the one hand, it is possible that the teaching-related stressors investigated in the present study are less salient in PE pre-service teachers because other stressors specific to their career stage are more important and, thus, more salient. For example, PE pre-service teachers have to deal with a number of additional formal (e.g., being evaluated by supervisors, oral exams) and developmental (e.g., developing a self-concept of being a professional PE teacher; Klusmann, Kunter, Voss, & Baumert, 2012; Kraler, 2008) demands. These additional demands (and also specific private demands of this age group; Ensign et al., 2018) were not assessed in our study but might be more important for pre-service teachers and, thus, influence their responses regarding the potential stressors that were assessed in the present study. On the other hand, the lower scores in PE pre-service teachers for potential stressors could be explained by the accurate preparation of their teaching. Pre-service (PE) teachers spend a lot of time in the preparation of their lessons (e.g., due to exams), and their preparation is—at least partly—supported by supervisors. This accuracy of preparation and the recourse to didactic methods recently gained in the preceding MA study program could lead to a structure of lessons which prevents the occurrence of some of the potential stressors. In particular, the process of precisely planning (didactic) methods may prevent the occurrence of lack of pupil discipline (e.g., classroom management; Jennings & Greenberg, 2009), inadequate facilities/equipment (e.g., seek for alternative equipment), lack of pupil motivation (e.g., autonomy support; Raabe, Schmidt, Carl, & Höner, 2019), and noise (e.g., classroom management; Jennings & Greenberg, 2009) and facilitate dealing with pupils’ heterogeneity (e.g., peer buddies; Laghi et al., 2016).

In turn, a lack of competence and experience in terms of preparation of and conducting teaching may be an explanation as to why PE student teachers reported a higher anticipated frequency of three specific potential stressors (lack of pupil discipline, inadequate facilities/equipment, lack of pupil motivation). In particular, the occurrence of these three potential stressors can be highly influenced by appropriate didactical methods. It is possible that the students have not yet gained sufficient experiences in didactic methods to prevent the occurrence of these stressors. Additionally, their first teaching experiences during the (long-term) internships of their study program could have been stressful because they were entirely new to them. While studying, there is limited time for practicing how to handle stressful situations that can occur while teaching. Before they enter their first internships in their BA studies, students did not have much time to try out useful skills and competences. In the practical semester with a long-term internship, MA students are then confronted with teaching for the first time without having much practical experience in dealing with daily demands. Taken together, this could have led to a potentially biased, exaggerated, and unrealistic expectation of future stressors in both BA and MA students, and this can also explain why the student groups do not differ from each other in potential stressors (except for motivation).

Higher reported frequency of noise in PE pre-service teachers and PE teachers compared to PE student teachers might be influenced by the overall exposition to this potential stressor. If PE student teachers have any teaching experience at all (e.g., due to an internship), other stressors and related teaching tasks will presumably have been more salient there (e.g., conflict-free lessons, motivating pupils). PE pre-service teachers and PE teachers, on the other hand, are confronted with noise on a daily basis and experience it continuously. This also fits with the finding of Sá et al. (2014) that the health impairments caused by noise among teachers depend on their working hours. However, there was no significant correlation between teaching hours and noise in our study.

Higher reported frequency of physical strain and lower reported frequency of problems with colleagues in regular PE teachers compared to PE student teachers can be explained by the teachers’ age. When including age as a covariate, the differences disappeared. This indicates that the occurrence of physical strain might be influenced by the PE teachers’ physical condition, highlighting the need for PE teachers to maintain their physical fitness until their retirement. The lower occurrence of problems with colleagues with growing age might be influenced by teachers’ personality development and growing status among colleagues over time. This effect is well-known from other work disciplines and life domains (e.g., Birditt, Fingerman, & Almeida, 2005). Older people are better at regulating interpersonal tensions than younger people.

Higher reported frequency of students’ heterogeneity in PE teachers compared to PE student teachers can be explained by the teachers’ academic education. The report of heterogeneity may be influenced by academic changes. In contrast to younger PE teachers, PE pre-service teachers or PE student teachers, many of the (older) PE teachers received less education in heterogeneity and inclusive learning during their study programs. However, given that heterogeneity and inclusion have become an important dimension of physical education during the past few years (e.g., due to law changes and due to societal changes), some PE teachers might still lack sufficient skills to work in an inclusive learning environment and to reduce the occurrence of potentially stressful situations. This explanation is in line with Lautenbach (2019), who found that there was no relationship between age and attitude towards inclusion in PE pre-service teachers (e.g., Jerlinder, Danermark, & Gill, 2010) but that younger PE teachers had a more positive attitude towards inclusion compared to older ones (Özer et al., 2013).

To sum up, the findings are in contrast to our hypothesis which was based on previous empirical findings in terms of perceived stress of (prospective) PE teachers (Schäfer et al., 2019) and based on the OST (Lawson, 1986; Richards et al., 2014, 2019). Considering OST, overall results show that potential stressors during PE lessons are more often reported in the phase of professional socialization (during PE university education) compared to the phase of organizational socialization (when entering school as a workfield). The results of the present study seem to be in contrast with the OST which emphasizes that the phase of organizational socialization is often perceived as a reality shock by (PE) pre-service teachers. However, our study assessed *potential* teaching-related stressors and not *actual* stressors and, thus, it is possible that the organizational socialization phase is indeed experienced as a reality shock when one is responsible for teaching on one’s own. Moreover, as outlined before, an accumulation of potential teaching stressors and additional stressor beyond teaching may lead to the reality shock and high perceived stress in (PE) pre-service teachers. Therefore, in the future, a differentiation of the OST for the German system should be considered (including a consideration of internships during studies and different areas of potential and actual stressors) and empirically tested.

### Strengths and limitations

There are three major strengths of our study: First, our study included a comprehensive set of teaching-related stressors among PE teachers. Second, our study was the first to investigate the (anticipated) stressors in three different career stages of PE teachers. Third, our study comprised a large sample size.

Despite these strengths, there are also limitations. First, a limitation exists regarding the interpretability of causality. Because our study was cross-sectional in nature and not based on any treatment, we cannot draw final conclusions about reasons for differences between career stages in terms of the stressors. Second, we have only examined a part of the entire stress process (according to the transactional model of stress and coping (Lazarus & Folkman, 1984, 1987) and to the model of teacher stress (van Dick, 1999)). For example, we have not explicitly assessed the appraisal of the potential stressors. Therefore, we do not know which potential stressors become actual stressors. As a consequence, it is unknown how PE teachers appraise the potential stressors’ controllability and, thus, how strongly the members of the different career stages suffer from the stressors (e.g., in terms of negative emotional reactions, impaired well-being or negative physical health consequences). In this regard, it is also important to consider that different stressors may accumulate and cause repeated bouts of negative emotions (Ensign et al., 2018; McCaughtry et al., 2006). Related to this, third, it is important to mention that the questionnaire used is inconsistent in a very few items. While the vast majority of items simply ask for the frequency of described stressors without including a stress appraisal (e.g., “Most of the pupils are absent-minded during PE”), few items include such a stress appraisal (e.g., the item “Important agreements with colleagues take place too late” includes a negative appraisal). Strictly speaking, all items would have to ask for potential stressors without including an appraisal. Future studies should therefore adjust the inconsistent items. Fourth, our instrument did not include stressors that are specific to the career stage of PE pre-service teachers. Fifth, the internal consistency of the factor physical strain (α = 0.28) of the ABIS questionnaire was very low. However, even in the initial validation study of the ABIS (Heim & Klimek, 1999), physical strain showed low internal consistency (α = 0.52). Further studies working with the ABIS noticed similar results and authors argue that the low internal consistency is caused by the low number of items and the heterogeneity of item content which is nevertheless necessary for a comprehensive measurement of physical strain (Miethling & Brand, 2004; Miethling & Sohnsmeyer, 2009).

## Conclusion

Based on the findings of the present study, several recommendations for practical application can be derived. These recommendations relate to the individual level (i.e., the (prospective) PE teacher) and to the organizational level (i.e., the school system, the university system) both of which are linked to each other. In terms of the individual (prospective) PE teachers, there is a need for (proactive) coping interventions that are tailored to the stressors which are salient in a specific career phase which was also suggested by other authors before (Richards, Housner, & Templin, 2018; Ziert, 2012). These interventions should contain didactic methods as active coping strategies as well as psychological coping strategies. First of all, all (prospective) PE teachers should be sensitized to potential stressors they will face during their daily routine as PE teachers. In this regard, possible individual stress reactions should be made subject of discussion. All (prospective) PE teachers should then learn psychological strategies (e.g., acceptance) which aim at coping with potential stressors that can only be controlled to a limited extent (e.g., noise, curriculum). When having a look at the career stages separately, regular PE teachers could be taught further knowledge and new didactic methods during programs of (regular) continuous professional development (e.g., Slingerland et al., 2021). This could, for example, facilitate dealing with heterogeneity. Aside from that, they should also maintain their physical fitness. For PE pre-service teachers, the major focus should be on dealing with the multiple areas of stressors they face (e.g., teaching-related stressors, non-teaching-related stressors in school, exams, seminar, developing a profession-related self-concept). For instance, in terms of the development of a profession-related self-concept, PE pre-service teachers might benefit from co-teaching (Blankenship & Coleman, 2009; Zach, 2020) and mentoring approaches (Ensign & Mays Woods, 2016). With the help of these approaches, they could teach in cooperation with experienced PE teachers and learn from their routines. Towards the end of the pre-service phase, the focus of these approaches should also be on dealing with the growing amount of teaching hours (which occurs at the latest with the completion of the pre-service phase) because this enhances the occurrence of teaching-related stressors. However, it must be ensured that the mentors fit the PE pre-service teachers (e.g., positive relationship quality between them). Often the pre-service teachers do not perceive a fit (Ziert, 2012). Regarding exams, it might be helpful that PE pre-service teachers learn a wide repertoire of psychological coping strategies for the flexible handling of different demands that might appear within the preparation phase for exams or during exams (e.g., handling time pressure). These strategies may also be helpful in a teacher’s everyday life. Tailored coping interventions for PE student teachers should contain didactic methods to deal with stressors, such as inadequate facilities (e.g., being able to modify exercises) or lack of pupil discipline (e.g., negotiating with students; Wahl-Alexander, Curtner-Smith, & Sinelnikov, 2018). Furthermore, PE student teachers might also benefit from a wide repertoire of psychological coping strategies to prepare them for a flexible handling of stressful situations which are new to them.

On the organizational level it is important to (further) offer and extend the interventions mentioned above and to implement measures for stressors that (prospective) PE teachers themselves can hardly influence. Policies and practices in the school system should implement measures to prevent PE teachers from stressors (see also Ziert, 2012). For instance, to reduce the impact of high noise it might be helpful, for example, to implement structural measures in the sport facilities that reduce the level of noise (e.g., constructional changes of gyms). With regard to the heterogeneity of pupils, measures such as team teaching or reduced class sizes might have a positive effect on the PE teachers stress reaction. In addition, teachers should be involved continuously in the revision of curricula to better adapt the curricula to the teaching reality. In the university system, curricula should also set a focus on improving stress regulation, including knowledge about potential stressors and stress reactions and the acquisition of strategies to cope with stressors and stress reactions. To date, this is often inadequately pursued in teacher education programs.

In future research, studies should deepen and extend the existing investigations on stressors. A deepening should take place by comparing PE teachers of different career stages on a longitudinal basis. In doing so, these studies should consider developmental effects (e.g., due to internships during the BA/MA study programs) and also consider stressors which are specific to a certain career stage (e.g., exams of PE pre-service teachers) or not related to teaching per se (e.g., meeting parents). Also, in addition to data gathered through self-reports of (prospective) PE teachers, objective data or data sources based on pupils’, principals’, and other teachers’ perspectives may contribute to gaining knowledge in this area.

An extension of the existing investigations should be made by including further group comparisons and additional psychological variables. First and foremost, studies should compare stressors between different types of schools. Due to the different developmental stages of the children, for example, differences between primary and secondary school can be expected. Furthermore, introducing additional variables, future investigations should take a closer look at the associations between teaching methods, psychological coping and organizational changes on the one hand, and stressors and emotional reactions to these on the other hand. These studies may, for example, examine how precisely planning (didactic) methods may prevent the occurrence of stressors such as lack of pupil discipline (e.g., classroom management; Jennings & Greenberg, 2009), inadequate facilities/equipment (e.g., seek for alternative equipment), lack of pupil motivation (e.g., autonomy support; Raabe et al., 2019) or noise (e.g., classroom management; Jennings & Greenberg, 2009) and how they may facilitate dealing with pupils’ heterogeneity (e.g., peer buddies; Laghi et al., 2016). Finally, studies testing the effectiveness of interventions targeting stressors should be investigated in randomized controlled trials.

## Footnotes

1 The sample was identical to that of Schäfer et al. (2019) in their study investigating perceived stress in (prospective) PE teachers, except for 10 persons who had missing data with regard to sources of stress and who were, thus, not included in the present study.

2 We did not compare BA vs. MA students separately because in the German system, the development paths of (PE) teachers are typically subdivided into three different phases. Also, the period of time of being a PE teacher is rather long compared to the period of time it takes to study. In order to compare the three phases we decided to look at the whole study time as one single phase. Moreover, additional control analyses that were carried out as a precaution showed only one significant difference in potential stressors between study programs (motivation: *F*(3, 714) = 108,39, *p* = < 0.001, η^2^ = 0.31; BA students > MA students, *p* < 0.001, *d* = 0.28); for all of the other stressors, comparisons were nonsignificant.

## Declarations

The study has been performed in accordance with the ethical standards laid down in the 1964 Declaration of Helsinki and its later amendments. Informed consent was obtained from all individual participants included in the study. The study was approved by the local ethics committee.
